# When and How-Long: A Unified Approach for Time Perception

**DOI:** 10.3389/fpsyg.2016.00466

**Published:** 2016-03-31

**Authors:** Michail Maniadakis, Panos Trahanias

**Affiliations:** Computational Vision and Robotics Laboratory, Institute of Computer Science, Foundation for Research and Technology HellasHeraklion, Greece

**Keywords:** time perception and timing, temporal distance, past perception model, when, how long, computational modeling, temporal cognition

## Abstract

The representation of the environment assumes the encoding of four basic dimensions in the brain, that is the 3D space and time. The vital role of time for cognition is a topic that recently attracted increasing research interest. Surprisingly, the scientific community investigating mind-time interactions has mainly focused on interval timing, paying less attention on the encoding and processing of distant moments. The present work highlights two basic capacities that are necessary for developing temporal cognition in artificial systems. In particular, the seamless integration of agents in the environment assumes they are able to consider *when* events have occurred and *how-long* they have lasted. This information, although rather standard in humans, is largely missing from artificial cognitive systems. In this work we consider how a time perception model that is based on neural networks and the Striatal Beat Frequency (SBF) theory is extended in a way that besides the duration of events, facilitates the encoding of the time of occurrence in memory. The extended model is capable to support skills assumed in temporal cognition and answer time-related questions about the unfolded events.

## Introduction

Our sense of time exhibits unique characteristics that distinguishes it from the typical group of human senses (sight, hearing, touch, smell, and taste). A crucial difference is that the sense of time is not associated with a specific sensory system in the brain. As it is noted in Bruss and Ruschendorf (2010), the perception of time seems different in nature from what we usually understand as perception. It seems to have its own ways and own laws. Since we cannot stop time, we cannot experience a moment twice. In the contrary, we can hear a sound, view a light, taste a food as many times as we want.

In an attempt to understand the unique characteristics of time perception, the recent years, a significant amount of research studies have been devoted on understanding the brain mechanisms that enable experiencing and processing time, with controversial theories attempting to explain experimental observations. Broadly speaking, there are two main approaches to describe how our brain represents duration (Ivry and Schlerf, 2008; Bueti, 2011). The first is the dedicated approach (also known as extrinsic, or centralized) that assumes an explicit metric of time. This is the oldest and most influential explanation on interval timing. The models included in this category employ mechanisms that are designed specifically to represent duration. Traditionally such models follow an information processing perspective in which pulses that are emitted regularly by a pacemaker are temporally stored in an accumulator, similar to a clock (Gibbon et al., 1984; Droit-Volet et al., 2007). This has inspired the subsequent pacemaker approach that uses oscillations to represent clock ticks (Miall, 1989; Large, 2008). Following a broader consideration, the Striatal Beat Frequency (SBF) model assumes timing to be the results of the coincidental activation of basal ganglia neurons by cortical neural oscillators (Matell and Meck, 2004; Meck et al., 2008). Other dedicated models assume monotonous increasing or decreasing processes to encode elapsed time (Staddon and Higa, 1999; Simen et al., 2011). The second approach includes intrinsic explanations (also known as distributed) that describe time as a general and inherent property of neural dynamics (Dragoi et al., 2003; Karmarkar and Buonomano, 2007). According to this approach, time is intrinsically encoded in the activity of general purpose networks of neurons. Thus, rather than using a time-dedicated neural circuit, time coexists with the representation and processing of other external stimuli. Recent models combine intrinsic and dedicated representations into active oscillations that do not only produce “ticks” but additionally adjust their characteristics to perceive, measure, and process time in order to facilitate the accomplishment of a variety of temporal tasks (Maniadakis and Trahanias, 2015). Similarly, models assuming oscillations with adaptive pulse rates extent the classic pacemaker-accumulator model to accomplish timescale invariance in interval timing (Simen et al., 2013).

The aforementioned models focus on estimating the duration of events (i.e., *how-long*), without typically paying much attention on the time of occurrence of events (i.e., *when*), as an important temporal information. The combined consideration of these two temporal aspects is vital for understanding the evolved phenomena in the environment in a rich and meaningful way. While interval timing is typically related to short-term time perception, considering *when* events have occurred is mostly related to the perception of mid and distant past. It is now believed in the timing community that the short-term duration perception mechanisms in the brain are different than those involved in the long-term, past perception (Aschoff, 1985; Rammsayer, 1999; Lewis and Miall, 2003).

However, given that the present is included in the entire timeline linking the past and the future, it is reasonable to assume a connection between the short- and long-term time perception. Along this line, the present work investigates the possibility that a universal time source may support both aspects of time perception. This is the focus of the present study which explores possible means for combining *when* and *how-long* in a single cognitive system. This does not aim to argue that the two mechanisms coincide or overlap. The subsystems of short- and long-term time perception are kept separate but it is possible that they share common timing inputs and in that sense we are interested to explore their possible bridging. It is noted that in order to explore the long- and short-term aspects of time perception, the implemented models must consider both the moments experienced during the occurrence of events and the moments passing without being associated to the given event. These two time periods exhibit very different characteristics as we will discuss in the following sections.

The present work adopts a memory encoding perspective to explore the possible mechanisms supporting *how-long* and *when* temporal cognition. Interestingly, besides providing an explanation on how the two times related cognitive capacities may be linked, the present work accomplishes a crucial milestone for introducing time perception in artificial systems enabling the later to consider the inherent temporal dimension of human-machine symbiotic interaction.

The composite model is developed following an incremental procedure. We start by implementing a neural network model that is capable to estimate and memorize the duration of simple tone-events. The model is implemented using a “black box” artificial coevolutionary procedure that tunes system components and enforces their cooperation. Subsequently, we consider the possibility of extending the model with the ability of keeping track the time of occurrence of the underlying events. We explore whether the previously implemented mechanism of time flow perception that is used for interval timing can be also employed for encoding when events have occurred. Moreover, we explore the option that past perception may use temporal distance measures as indicators of past times. Our experiments show that a single time source can facilitate encoding of both the duration and the time of occurrence of events.

## Artificial evolution of interval timing model

To develop a brain inspired duration perception system, we borrow ideas from the Striatal Beat Frequency (SBF) model (Matell and Meck, 2004; Meck et al., 2008) that is one of the most widely referenced paradigm explaining interval timing in the brain. The model assumes that durations are coded by the coincidental activation of a large number of cortical neurons projecting onto spiny neurons in the striatum that respond to timing patterns. The present work explores a very simple version of the SBF model using only a small number of input oscillatory signals. The goal of this simplified model is not to compete against the original SBF model, but rather to suggest a new direction for using interval timing models.

### Modeling

We employ the coevolutionary neural network framework that has been described in detail in (Maniadakis and Trahanias, 2008, 2009) to develop a modular neural network system for interval timing. In the past, we have used the same technology to develop cognitive models for artificial agents, which have been capable of time-informed behavior switching (Maniadakis et al., 2009) and multi-context duration processing (Maniadakis and Trahanias, 2015).

**Figure 1:**
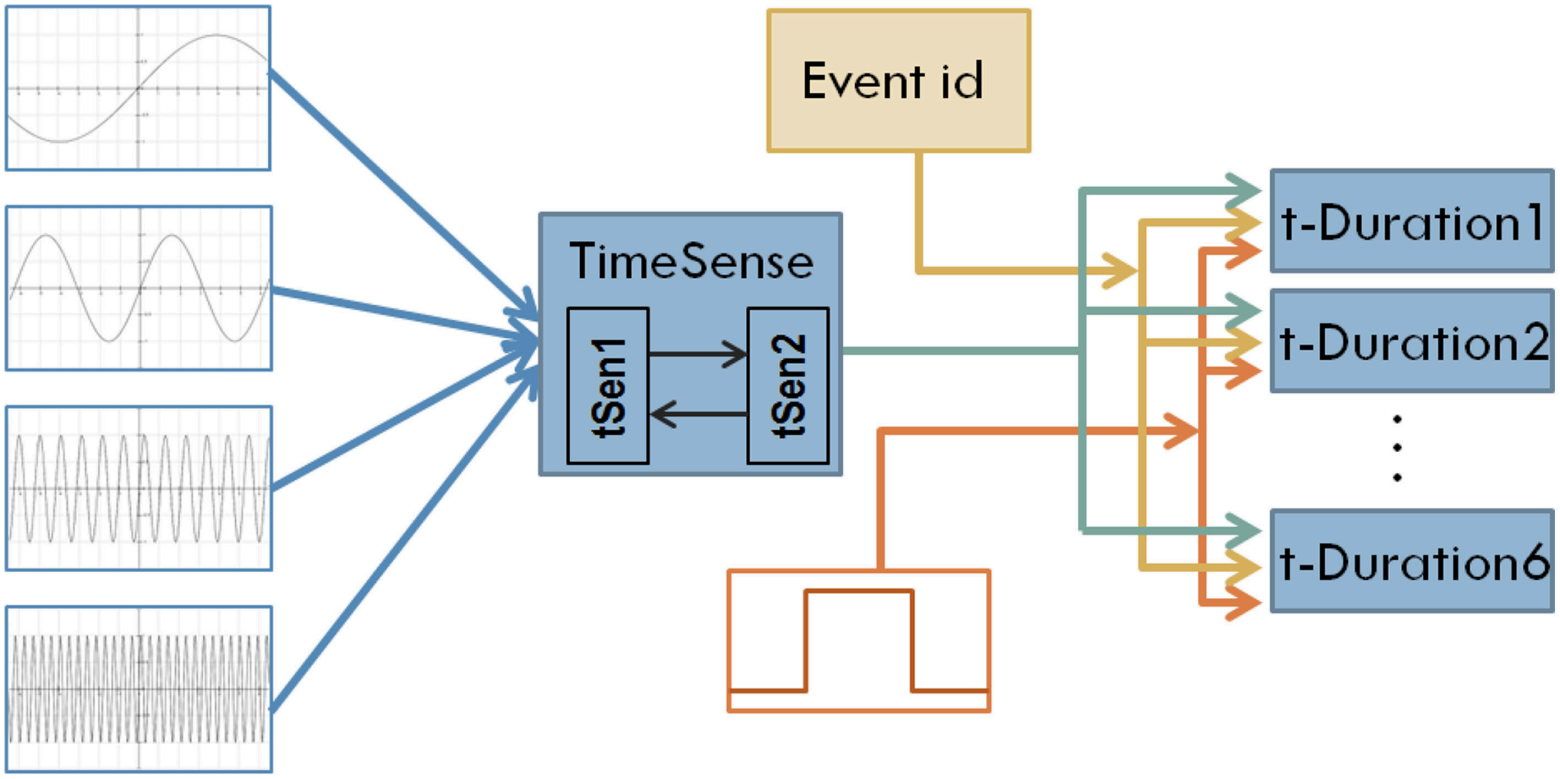
**The structure of the first model of interval timing**. A recurrent TimeSense module blends oscillatory signals to develop the filling of flowing time, properly formulated to enable interval timing in the modules t-Duration1 …t-Duration6 that are also fed by the external tone signal and the id of the perceived event.

The structure of the neural network model is shown in Figure 1. In short Continuous Time Recurrent Neural Networks (CTRNNs) are used as modules to develop a composite cognitive system. CTRNNs represent knowledge in terms of internal neurodynamic attractors and it is therefore particularly appropriate for implementing cognitive capacity that is inherently continuous, similar to time perception. The neurons implementing CTRNN components are governed by the standard leaky integrator equation:

(1) $$\frac{\text{d}\gamma_{\text{i}}}{\text{dt}}=\frac{1}{\tau }\left(-\gamma_{\text{i}}+\sum_{\text{k }=\;1}^{\text{R}}{\text{w}}_{\text{ik}}^{\text{s}}{\text{I}}_{\text{k}}+\sum_{\text{m }=\;1}^{\text{N}}{\text{w}}_{\text{im}}^{\text{p}}{\text{A}}_{\text{m}}\right)$$

where γ_*i*_ is the state (cell potential) of the i-th neuron. All neurons in a network share the same time constant τ = 0.25 in order to avoid explicit differentiation in the functionality of CTRNN parts. The state of each neuron is updated according to external sensory input *I* weighted by ws, and the activity of presynaptic neurons A weighted by w^p^. After estimating the state of neurons based on the above equation, the activation of the i-th neuron is calculated by the non-linear sigmoid function according to:

(2) $${\text{A}}_{\text{i}}=\frac{1}{1+{\text{e}}^{-(\gamma_{\text{i}}-\theta_{\text{i}})}}$$

where θ_*i*_ is the activation bias applied on the *i-th* neuron. The model considered in the present study assumes 16 neurons for the building blocks tSen1, tSen2, and 2 neurons for the blocks implementing t-Duration1, …, t-Duration6. A hierarchical coevolutionary procedure is used as a mechanism for tuning CTRNN modules, specifying synaptic weights and activation bias of neurons.

Following the assumption of fusing cortical neural oscillators for implementing sense of time, we use four oscillatory signals at different frequencies as inputs to the model. The use of such a small number of oscillatory inputs keeps manageable the complexity of the model providing at the same time the opportunity to obtain insight in the dynamics self-organized internally in the CTRNN. The oscillatory signals used in the current study are as follows:

(3) $$\begin{array}{l} {\text{Inp}}_{1}=\text{sin}(\text{4t}+{\text{k}}_{1})+u(-0.05,0.05)\\ {\text{Inp}}_{2}=\text{sin}(\text{t}+{\text{k}}_{2})+u(-0.05,0.05)\\ {\text{Inp}}_{3}=\text{sin}(0.25\text{t}+{\text{k}}_{3})+u(-0.05,0.05)\\ {\text{Inp}}_{4}=\text{sin}(0.1\text{t}+{\text{k}}_{4})+u(-0.05,0.05) \end{array}$$

Parameters k_1_, k_2_, k_3_, k_4_ ϵ [0,π], implement random time shifts initialized at the beginning of every experimental session (i.e., different values are assumed for each evolutionary run, see below). Additive noise implemented as a uniform distribution in the range [–0.05, 0.05] aims to improve generalization of the internal representation of time and thus enable robust and accurate duration estimation.

Each temporal moment processed by the model is associated to one simulation step. Interestingly, assuming that one simulation step corresponds to 5–10 ms, the input signals described in Equation (3) can be associated to the known frequencies of cortical neural oscillations, from the 1–4 Hz of the delta band, up to the 30–70 Hz for the gamma band. It is noted that, for years, it is has been hard to identify a single frequency band dominating temporal processing, (Treisman, 1984; Wiener and Kanai, 2016). However, modern approaches assume that the combination of bands might be the key for explaining sense of time (for a discussion, see Kononowicz and van Wassenhove, 2016 in the present Research Topic). Such an assumption provides added value to our model, which combines oscillations at very different frequencies to develop sense of time. However, the input signals considered in the present study were not originally designed with cortical oscillation bands in mind, and thus we would like to avoid building further on this assumption. Besides targeting interval timing in the range of a few seconds, the model does not assume an explicit correspondence between simulation steps and the known metrics of physical time (e.g., ms, or sec). The main goal of the present work has been the development of a brain-inspired time perception system for robotic agents engaged in long-term symbiotic interaction with humans.

Turning back to Figure 1, oscillatory inputs project into a composite TimeSense module consisting of two recurrently connected sub-modules. The TimeSense module aims at gradually transforming oscillatory inputs to a composite time flow representation that is adequate for interval timing. To facilitate the applicability of the model in robotic applications, we use working memory to store the temporal properties of a small number of recently experienced events. In the current implementation we explore scenarios assuming the random occurrence of six events (the capacity of working memory) in a session of 1000 simulation steps. We employ 6 different duration estimation modules each one devoted to the perception of one tone-event. The duration estimation modules receive a binary tone input that represents the occurrence of events. Tones have randomly specified lengths that represent the duration of events. A binary signal representing the unique ID of the event enables differentiating the measured interval lengths. The actual duration of events is randomly specified every time a NN model is loaded and tested. We enforce a minimum distance of 100 moments between consecutive events.

### Parametric tuning

The training of the model is achieved using Hierarchical Cooperative CoEvolution as described in (Maniadakis and Trahanias, 2008, 2009). By using this “black box” coevolutionary scheme we are able to consider the specialized characteristics of each component in the model and additionally enforce their synergetic functionality to accomplish the desired overall performance for the composite system.

We assume a brain-like encoding of interval timing. More specifically, a ramp-like encoding of time has been identified in the brain of monkeys (Leon and Shadlen, 2003; Maimon and Assad, 2006; Mita et al., 2009) for durations up to a few hundreds of milliseconds. The proposed model abstracts these findings by implementing a similar ramping mechanism for short-term interval timing, aiming mainly to support robotic applications.

Error-based functions are used to evaluate the performance of each event-specific module tDur1,…, tDur6. In particular, the desired output of the module associated to the *i-th* event starting at time *st*_*i*_ and finishing at time *e*_*i*_, having a maximum duration *M* (i.e., *e*_*i*_−*st*_*i*_ < *M*) equals to:

(4) $$D_{i}\left(t\right)=\{\begin{array}{ll} \;0,\; & t<st_{i}\\ \left(t-st_{i}\right)/M, & st_{i}\leq t<e_{i}\\ \left(e_{i}-st_{i}\right)/M, & e_{i}\leq t \end{array}$$

In the current study we investigate events with maximum duration *M* = 50 moments. The function that measures the success of the *i-th* temporal duration module is:

(5) $$EDur_{i}=\sum_{t}{\left(out_{i}(t)-D_{i}(t)\right)}^{2}$$

This is the key component of the fitness function *ff*_*i*_ that drives the evolution of the corresponding module accomplishing parameter tuning:

(6) $$ff_{i}=\frac{1000}{EDur_{i}}$$

Higher values of *ff*_*i*_ indicate better performance of the *i-th* duration module.

To accomplish parametric tuning for the neural network modules representing the components t-Sen1 and t-Sen2, which have a supportive role for all duration estimation modules, we employ a mixture of the afore mentioned fitness functions, described by:

(7) $$ff=\prod_{i}ff_{i}$$

The hierarchical cooperative coevolutionary procedure (Maniadakis and Trahanias, 2008, 2009) accomplishes parametric tuning and optimization of component modules, enforcing their collaborative performance toward a successful composite model. We use one population of 1000 artificial chromosomes for each CTRNN module considered in the model. Each chromosome, encodes a different configuration of the module. We combine candidate module configurations to develop full configurations of the complete system, which are tested on the duration estimation task described above. The 20% of the best performing chromosomes in each population are selected for reproduction following single point crossover. Mutation is applied on new chromosomes with a probability 2% for each encoded parameter. Mutation is implemented as additive noise in the range [–10%, 10%] relative to the previous value of the parameter.

### Results

**Figure 2:**
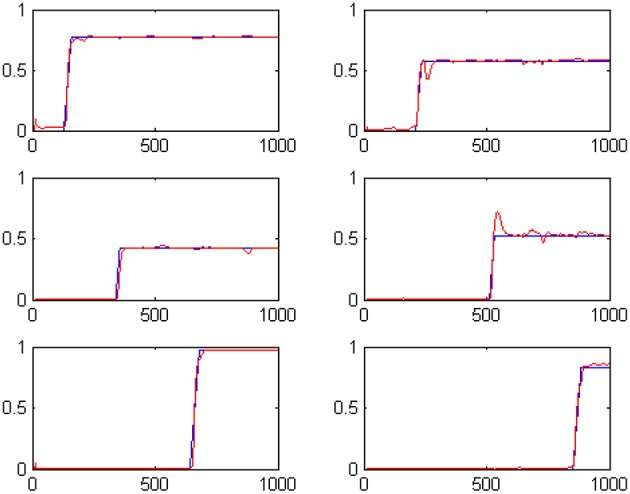
**The outputs of the six t-Duration modules which are responsible for measuring duration of six different tone events with randomly specified durations**. Blue lines represent desired outputs, while red lines show actual system output.

We have evolved the above described coevolutionary scheme for 500 generations, producing a successfully tuned CTRNN model for interval timing. An indicative set of results for six randomly specified tone events is shown in Figure 2. The fact that numerous event durations can be simultaneously preserved in the system is a valuable addition to interval timing models that enables further processing of the memorized durations. In particular, it has been straight forward to use a Multi-layer Perceptrons (MLPs) to develop decision making systems capable of comparing any two of the memorized durations to accomplish duration comparison tasks similar to those studied in (Droit-Volet et al., 2010).

**Figure 3:**
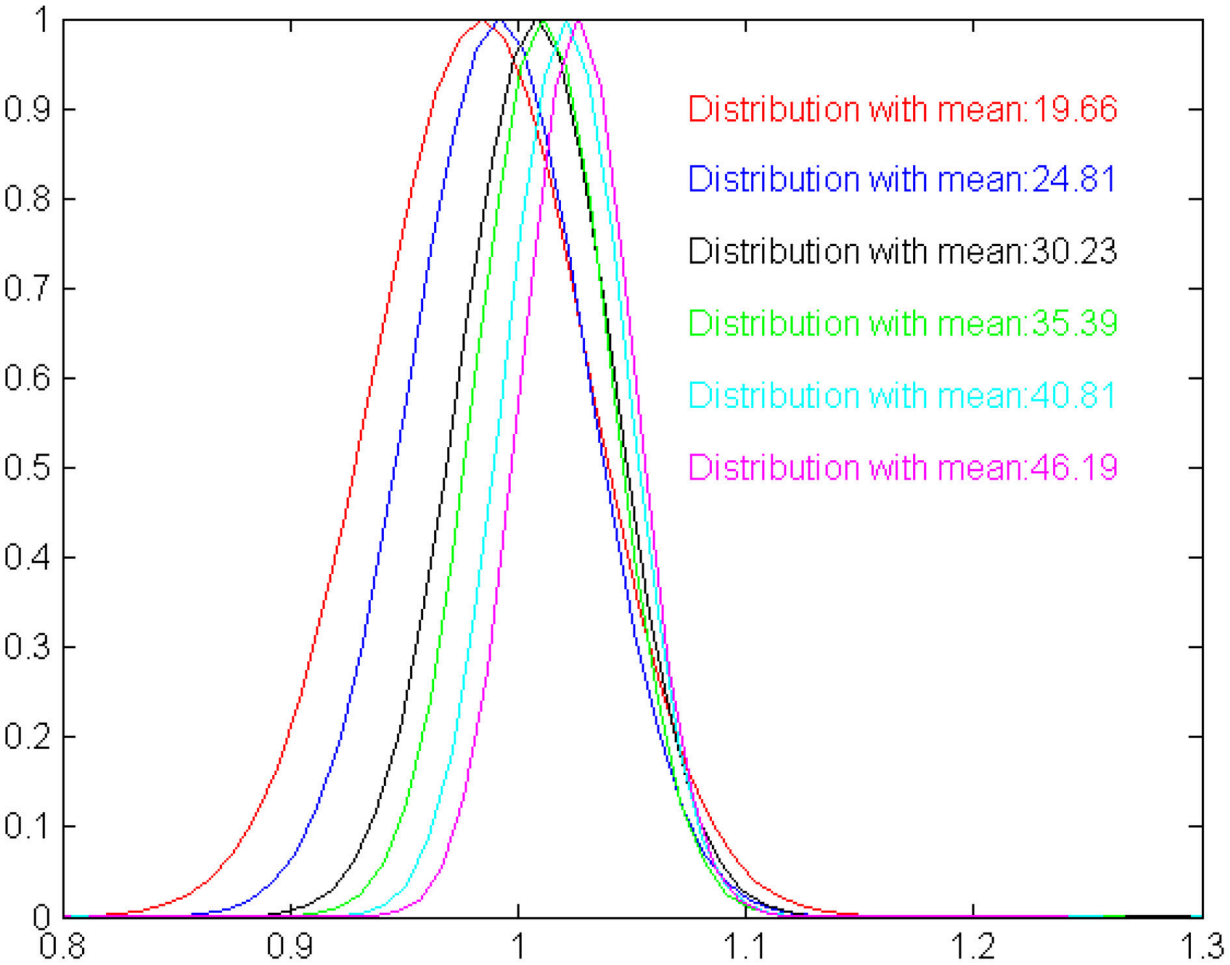
**A graphical illustration of the time estimation distributions shown in Table 1, scaled by the expected duration means**. The more the distributions are identical the more the model is compatible with the scalar property. For our model, estimated means are slightly shifted against the expected values, and standard deviation increases slower than expected by the Weber law.

Besides extensively testing the model with randomly specified interval times up to *M* simulation steps, we explore whether the output of the model exhibits the scalar characteristics that are typical observed in biological timing mechanisms (Lejeune and Wearden, 2006). Scalar timing implies that (i) measurements should vary linearly and near-accurately as time increases and (ii) the variance of perceptual mechanism increases as the duration of time also increases. To get an estimate of the scalar characteristics of the model, we have studied its ability to correctly estimate durations of 20, 25, 30, 35, 40, and 45 moments (without this limiting the model to perform successfully for in-between durations). For each one of the six durations considered here, we perform 50 statistically independent runs, feeding the model with randomly initialized oscillatory inputs. The mean and standard deviation for each one of the durations considered are shown in Table 1. Clearly, the average of the estimated intervals remains close to the true time in all cases, satisfying mean accuracy. The variance increases as the model experiences longer intervals, however, in a rate that is slower to the increase of the mean. The scalar property assumes a constant coefficient of variation (the ratio of the standard deviation to the mean), which is not true for our model. This is depicted more clearly in Figure 3, where relevant output distributions are scaled by the expected duration value. Even if the model is not fully compatible with the scalar property, Table 1 shows that the output of the model is sufficiently accurate for making the model usable in robotic systems. Nevertheless, it is worth emphasizing that, currently, the two main characteristics of the scalar property have been self-organized without any explicit instructions by the modeler. Therefore, it seems valid to assume that our model can be easily rendered fully compatible to the scalar property, by introducing a constraint for a constant coefficient of variation in the fitness function of the evolutionary design procedure.

**Table 1:** **Studying the scalar properties of the model**.

**Actual time**	**20**	**25**	**30**	**35**	**40**	**45**
Estimated time—Mean	19.66	24.81	30.23	35.39	40.81	46.19
Estimated time—STD	0.87	0.93	1.04	1.09	1.17	1.21

The model does not assume a direct relationship between simulation time and physical time. The number of simulation steps is used as indicator of the elapsed time.

The notably small variations in time estimations shown in Table 1 (we remind it summarizes the results of 50 randomly initialized runs of the model) indicate that the implemented model is particularly tolerant to the noise added in the oscillatory input. To further assess model robustness, we have explored the performance of the model against different levels of input noise. Results are summarized in Table 2. The model shows to perform satisfactorily for input noise up to the range [–0.07, 0.07]. More noise than that significantly affects the estimation of durations for specific events. In particular, noise in the range [–0.09, 0.09] often results into a single mismeasured event, noise in the range [–0.11, 0.11] results into more than two mismeasured events (on average 2.3), and noise in the range [–0.13, 0.13] results, into nearly random duration measurements. Practically, the increase of noise affects the performance of the TimeSense modules which in turn introduces disturbances (i.e., occasional picks) in the corresponding ramping activities therefore destroying accurate interval timing.

**Table 2:** **Model performance against different levels of input noise**.

**Noise range**	**[−0.03, 0.03]**	**[−0.05,0.05]**	**[−0.07,0.07]**	**[−0.09,0.09]**	**[−0.11,0.11]**	**[−0.13,0.13]**
Estimated time average error	0.47	0.53	0.72	6.38	10.13	18.67

Interestingly, in the case that the noise is added to the input signal for a relative short time (e.g., < 10 simulation steps) the performance of the model remains largely unaffected, even for noise in the range [–0.13, 0.13]. This is explained by the use of leaky integrator neurons which smooth out the strong but temporally-short noise, enabling the model to quickly recover into the normal mode of operation.

While previous SBF models have been particularly sensitive to sensory noise (Matell and Meck, 2004; Gu et al., 2015), the model implemented in the current work exhibits more robust performance, therefore enabling interval timing in noisy environments. This is a particularly desirable feature that is developed for free in the model due to the noise included in the oscillatory sensory inputs and the randomness introduced in the experimental setup. This is mainly because we do not artificially describe coincidental activation of oscillatory inputs, but we let the neural network self-organize the fusion of inputs. Fitness assignment favors the more robust neural networks which filter out noise and estimate durations that are closer to the target. Therefore, the evolutionary procedure produces solutions that are gradually more tolerant to noise. However, it is worth emphasizing that sensory noise has been shown to facilitate time scale invariance in the case of a large number of input neural oscillators (Oprisan and Buhusi, 2014).

## Extend the model to address “when”

The model described above has been able to accomplish accurate interval timing in a series of randomly initialized binary events. With this timing mechanism at hand it is particularly interesting to explore, whether we can achieve other temporal cognition skills beyond interval timing. In the current study we explore if it is possible to use the previously developed timing mechanism as a base for encoding information related to the time of occurrence of events, that is to represent time moments in the distant past. While estimating the duration of an event requires the active percepton of the external stimulus, keeping track of *when* that event occurred assumes perceiving time that is not anymore related to the underlying event, filtering also out any other external input that may appear in the meanwhile. This is an important qualitative difference that distinguishes *when* and *how-long* perception.

There are two alternative options for encoding when an event has occurred in the past. The first assumes a coordinate system centered on “now,” e.g., “John was here one hour ago.” Following this approach the center of the coordinate system is non-static but it is moved together with the flow of time, causing a continuous increase in the time elapsed from the occurrence of the event until now (i.e., in a while, the above statement will change to “John was here two hours ago” and so on). The other alternative assumes a timeline centered on a predefined moment that is assumed to represent the zero-point and all time moments are perceived relative to that particular zero-point. For example, most western cultures assume as zero-point the birth of Jesus Christ and thus dates are typically measured as distances from this point, e.g., “I met John on February 10, 2015”.

Human adults can equally perceive both alternative options. However, it seems more likely that the development of the past perception for young children starts centered on “now”. This is because even if infants are capable to perceive time very early in their life (Droit-Volet, 2011), the conceptual development of an objective zero point develops not earlier than the middle childhood (Friedman, 2005). The now-centered perception of time is further supported by developmental studies showing a decline in the accuracy of children responses with increasing distances to the past (Friedman, 1998) and the fact that children have autobiographical memories before they learn how to use clocks and calendars (Campbell, 1997). Finally, from a numerical point of view, young children seem to slowly develop the concept of ordinal relationship between small values which gradually develops to the understanding of the broader number line (Gallistel and Gelman, 1992; Rouder and Geary, 2014). The above suggest that a first, basic approach for representing when events have occurred should be implemented relative to “now” rather than relative to a fixed point in time. The latter option may be developed at a following stage as a higher level capacity that processes encoded events.

Interestingly, the now-centered representation of the timeline suggests that the duration perception mechanisms may have a key role in the representation of past times. To elaborate further on this assumption, we borrow from the past perception literature (Arzy et al., 2009; Wyer et al., 2010) the term “temporal distance,” which describes the temporal properties of past events in relation to the present. We implement the computational analogous of temporal distance in our model, and we investigate the possibility of using this measure as a representation of when events have occurred in the past.

In particular, we extend the model discussed in Section Artificial Evolution of Interval Timing Model to additionally incorporate the capacity of memorizing the times of events' occurrence based on the assumption of encoding temporal distances to the present. In that sense, the composite model will work in two different time scales (i) up to 50 simulation steps for the *how-long* mode and (ii) up to 1000 simulation steps for the when mode. The revised model will be capable of using a single sense of time to derive both the duration of events and their time of occurrence.

**Figure 4:**
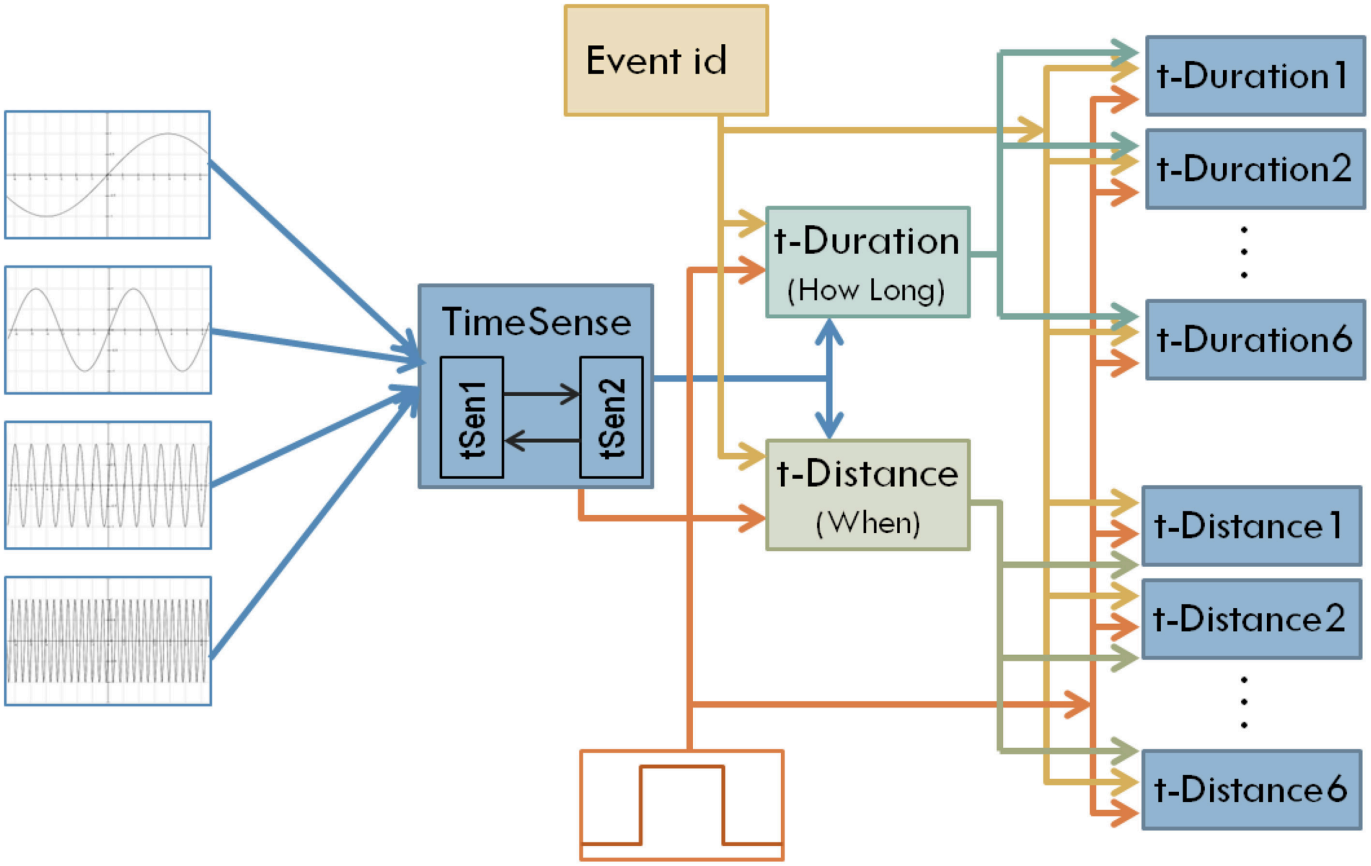
**The structure of the enhanced model that aims to address both interval timing and past time perception**.

Interestingly the coevolutionary framework used in the current work is particularly appropriate for the incremental modification and enhancement of modular neural network models (Maniadakis and Trahanias, 2009). To incorporate distant time perception, a set of neural network components is integrated into the model as shown in Figure 4. Two central components aim to transform general purpose sense of time to a form that is appropriate for measuring duration (t-Duration module) and temporal distance (t-Distance module). Similar to the earlier version, we use dedicated modules t-Duration1, t-Duration2 …t-Duration6 to memorize durations and modules t-Distance1, t-Distance2 …t-Distance6 to memorize temporal distances for the six tone-events considered in the current experimental setup. The CTRNN-based implementation of the modules assumes 16 neurons for the building blocks tSen1, tSen2, t-Duration, t-Distance, and 2 neurons for the blocks implementing t-Duration1, …, t-Duration6, and t-Distance1, …, t-Distance6.

A key issue for implementing temporal distances regards the representation of time in the long-term. There is classical debate on psychophysics asking whether the humans perceive the time-line in a linear or a logarithmic basis. Without any restriction^1^ the present work adopts the assumption of a logarithmic representation of distant time which is supported by recent experimental data (Arzy et al., 2009; Glicksohn and Leshem, 2011) and is in line with modern numerical cognition theories (Nieder and Miller, 2003). Cognitive models assuming logarithmic and other non-linear forms of time perception have also appeared in the literature (Staddon and Higa, 1999; van Rijn et al., 2014).

Following the logarithmic representation, the temporal-distance *TD* between current time *t* and the time *st*_*i*_ that the *i-th* event started, is encoded as:

(8) $$TD_{i}\left(t\right)=\{\begin{array}{ll} 0, & t\leq st_{i}\\ \text{log}(\frac{t}{st_{i}}), & st_{i}<t \end{array}$$

We use *TD*_*i*_(*t*) as the target of the *i-th* t-Distance module. Therefore, to evaluate the performance of the module encoding temporal distance of the *i-th* event we use an error-based measure that is:

(9) $$EDist_{i}=\sum_{t}{\left(out_{i}(t)-TD_{i}(t)\right)}^{2}$$

This is used to define the fitness function that drives the evolution of the corresponding *i-th* t-Distance module. In particular, the modules t-Distance1, t-Distance2, …t-Distance6 and all relevant incoming links are evolved according to the fitness function:

(10) $${\text{ff}}_{\text{dist},\text{i}}=(1000/{\text{EDist}}_{\text{i}})$$

Similar to the early setup of the coevolutionary procedure the modules t-Duration1, t-Duration2 …t-Duration6, and all incoming links are evolved according to the fitness function:

(11) $${\text{ff}}_{\text{dur},\text{i}}=(1000/{\text{EDur}}_{\text{i}})$$

The module specific fitness functions are properly mixed to develop composite fitness functions that drive the evolution of the supportive modules. More specifically, the fitness function of the module t-Distance considers the performance of all six t-Distance*i* modules:

(12) $${\text{ff}}_{\text{dist}}=\prod_{\text{i}}{\text{ff}}_{\text{dist},\text{i}}$$

Similarly, the fitness function of the t-Duration module considers the performance of all six t-Duration*i* modules:

(13) $${\text{ff}}_{\text{dur}}=\prod_{\text{i}}{\text{ff}}_{\text{dur},\text{i}}$$

Finally the root components of the system t-Sen1 and t-Sen2 that implement time sense are evolved according to both the temporal distance and the temporal duration criteria, resulting into the fitness function:

(14) $${\text{ff}}_{\text{global}}={\text{ff}}_{\text{dur}}*{\text{ff}}_{\text{dist}}$$

The hierarchical coevolutionary procedure accomplishes parametrical tuning of all system components taking into account their special features as well as the successful functionality of the composite time processing system. The hierarchical and synthetic structure of the fitness functions enforces the coevolutionary scheme to improve collaboration between the component neural networks. As a result, the coevolutionary procedure can successfully converge to partial solutions that synthesize a composite system capable of memorizing the duration and the time of occurrence of events.

### Results

**Figure 5:**
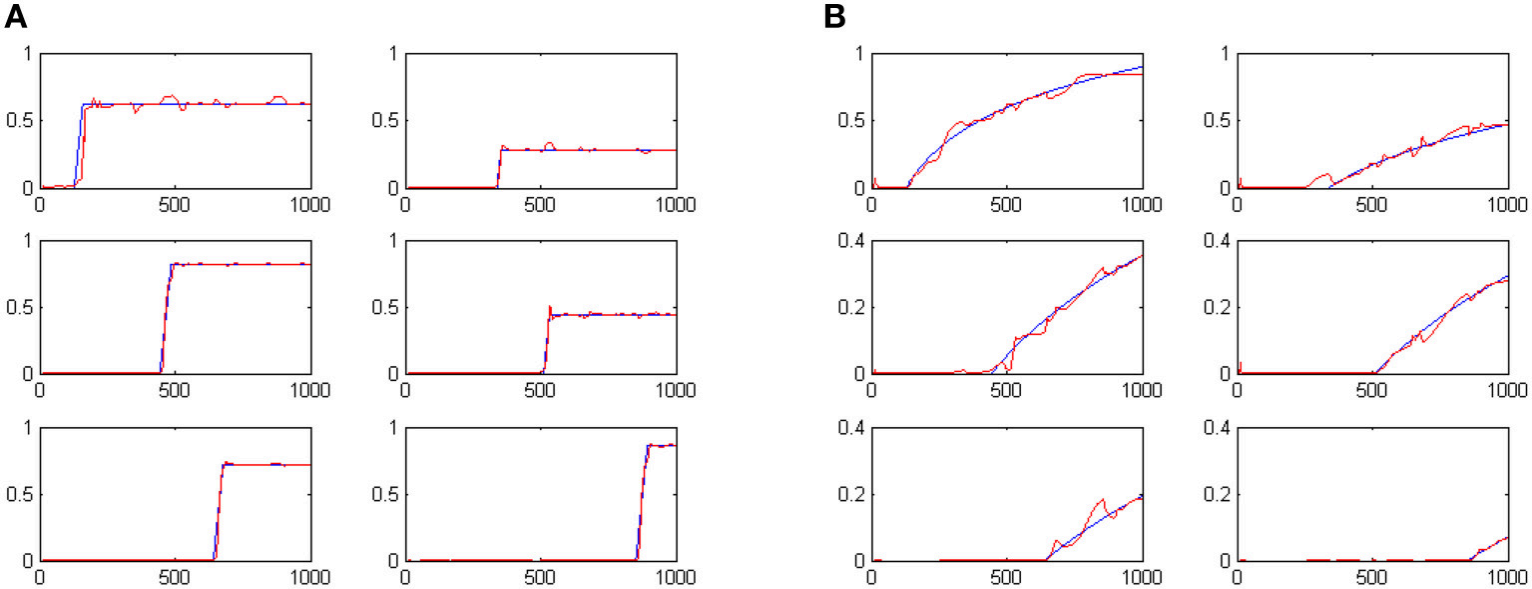
**System outputs after the perception of six random tone events**. Panel **(A)** shows the estimated duration of events. Panel **(B)** shows perceived temporal distance to the present as a means of representing when events have occurred.

Following the coevolutionary procedure described above, the cognitive system described in Section Artificial Evolution of Interval Timing Model is advanced to address both the *when* and the *how-long* aspects of events. The configurations of previously existing CTRNN modules have been reloaded and evolved further, together with the configurations of the newly introduced components. The extended cognitive system has been evolved for 300 epochs producing a composite cognitive system that can successfully process temporal information. Sample results of the system outputs when memorizing 6 randomly initiated tone events are shown in Figures 5A,B. The plots show in blue the desired output and in red the actual output of the system. For example the two plots shown in the first column, second line of Figures 5A,B encode the fact that a tone event of duration 42 (note: 42/50 = 0.84) has occurred at a past time that is 557 moments back from the present (note: log(1000/443) = 0.353).

**Figure 6:**
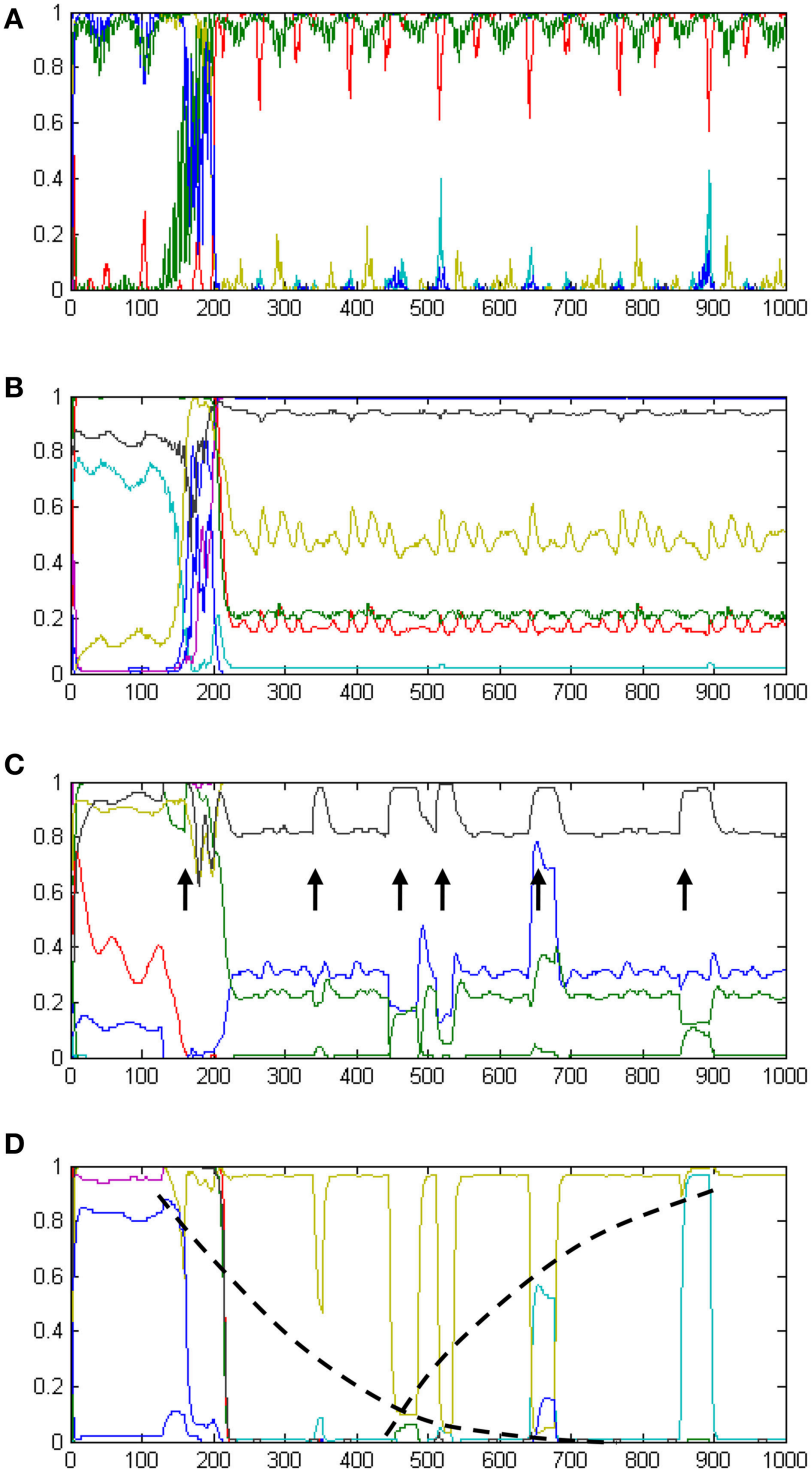
**A summary of the internal dynamics (neural activations) developed in the model over time**. Panel **(A)** shows neural activation in tSen1, the first receiving component of the recurrent TimeSense module. Panel **(B)** shows neural activation in tSen2, the second output component of the TimeSense module. Panel **(C)** shows neural activation in the t-Duration module which supports interval timing. Arrows indicate the times of event experiencing. The width of peaks is analogous to the duration of events, therefore enabling accurate duration estimation as shown in Figure 5A. Panel **(D)** shows neural activation in the t-Distance module which supports past perception. Neural activities shown in yellow and cyan implement internal time keeping of the elapsed time as illustrated by the log-shaped dotted black lines following their peaks.

The development of temporal processing internally in the model is shown in Figures 6A–D. The four plots show neural activity in the t-Sen1, t-Sen2, t-Duration, and T-Distance modules for the whole period of perceiving the 6 events. In the first stage of processing (Figure 6A), neural activity is mainly directed by the input oscillatory signals. Subsequently (Figure 6B) oscillations are mixed to produce a complex temporally structured neural activity. The first event occurs approximately at the moment 150. It seems that this event triggers a more structured oscillation fusion in t-Sen2 resulting in neural activity that looks like oscillation multiplexing. While the present model was not implemented on the basis of integrating oscillations that correspond to the known brain rhythms (delta band to gamma band), our results show that the combination of input signals at different frequencies may significantly contribute in the sense of time as suggested also in (Kononowicz and van Wassenhove, 2016).

At the third stage, processing separates to interval timing and temporal distance to the past. Neural activation in the t-Duration module is presented in Figure 6C. As it is shown in the plot, the length of the events appearing (approximately) at times 340, 440, 510, 650, and 860 is correlated to the width of the peak disturbances (marked with arrows), as shown in the respective plot. The final stage of processing is the one shown in Figure 5A, demonstrating the correct estimate of interval timing. Interestingly, longer durations correspond to flat peaks that take longer to smooth out, while shorter durations have no time to develop flat activities

Neural activation in the t-Distance module is shown in Figure 6D. The plot shows a gradual increase in the amplitude of activation disturbances as more and more events gradually occur. The dotted lines drawn on top of the neural activities shown in yellow and cyan reveal two non-linear measures to be kept internally in the model. The mixture of these two self-organized measures is adequate for measuring temporal distances to the present as it appears by the relevant outputs of the model in Figure 5B.

## Discussion

We have presented a neural network model that is capable of measuring short time intervals assuming linear ramp activity and keep track of past times based on the logarithmic representation of temporal distances. The model is implemented following a semi-automated procedure that assumes parameterized CTRNN modules attuned with the help of coevolutionary optimization. The tuning of model parameters is accomplished in an offline mode, similar to the supervised learning approach followed in other timing neural network models (Laje and Buonomano, 2013). Interestingly, evolutionary methods can be nicely combined with on-line adaptation procedures to facilitate life-long learning (Maniadakis and Trahanias, 2008) and thus enable modifying the range of processed durations.

The neuro-evolutionary framework considered in the present study provides increased flexibility in designing the internal mechanisms of the model, accomplishing to easily bridge oscillatory input and ramping activity in a single model. While the two mechanisms have been frequently considered contradictory in the literature, the use of oscillations with gradually adapted characteristics provides the basis for implementing effective interval timing mechanisms (Simen et al., 2013) and has been used for accomplishing multiple interval timing tasks (Maniadakis and Trahanias, 2015).

In contrast to previous works proposing timing models that have been rather minimally integrated with other cognitive functions (Gibbon et al., 1984; Staddon and Higa, 1999; Dragoi et al., 2003; Droit-Volet et al., 2007), the incremental NN modeling approach greatly facilitates the implementation of complex time-aware cognitive systems that will enable robotic systems to further exploit temporal cognition. The present work considers the strong coupling of time perception and short-term memory as suggested in (Gu et al., 2015). Other relevant works have considered spatiotemporal patterns related to motor behaviors (Laje and Buonomano, 2013). The use of spiking recurrent neural networks for timing has been shown to be particularly sensitive to noise (Banerjee et al., 2008). Relevant computational models shown that, especially for SBF, different types of noise may differentially affect the encoding and recall of timing intervals (Oprisan and Buhusi, 2014). Despite enforcing noise tolerance through learning (Laje and Buonomano, 2013), our study shows that the use of rate coding neurons may significantly facilitate model robustness.

The main contribution of the present study in comparison to the state of the art regards the use of past distance measures as a means of encoding the time of occurrence of experienced events. Our results show that a single timing source can be used as a basis for implementing cognitive systems capable of encoding *when* events occurred and *how-long* they have lasted. The proposed model suggests it is possible to bridge both short- and long- time keeping mechanisms that in the literature have been so far considered largely independent (Aschoff, 1985; Rammsayer, 1999; Lewis and Miall, 2003).

We note that the SBF-like characteristics assumed in the current implementation are not restrictive for bridging when and how long. Apart from the specific timing mechanism assumed by SBF, the proposed modeling approach could be nicely combined with other representations of time, such as (Miall, 1989; Staddon and Higa, 1999; Karmarkar and Buonomano, 2007; Large, 2008; Simen et al., 2011). However, even if nearly all timing models could equally support interval timing and past-distance measuring, using a single timing mechanism for both *when* and *how-long* can hardly comply with the brain studies explicitly distinguishing the two systems. Along this line, the current model assumes separate subsystems dedicated to the estimation of short-term durations and long-term temporal distances, providing the means to sufficiently address qualitative differences between them. This is accomplished by assuming different forms of temporal information to be readout by TimeSense neurons, which are subsequently processed assuming different mechanisms and processes.

The encoding of estimated times in memory highlights two very interesting problems that a time-aware cognitive system must concern in order to be functional in naturalistic conditions. The first problem regards how *how-long* and *when* should be represented in memory. In the former case the duration is necessary to gradually increase as long as the event is experienced by the agent and stop at a specific value that will be encoded in memory, representing the static (never changing again) duration of the event. The latter case assumes a counting mechanism that increases together with the evolution of the event but continues increasing after the end of the event, resulting into a dynamic (non-static) representation of past times relative to the present. The distinction between static and dynamic time representations gets even more complicated by considering the second problem, which regards how a cognitive system links specific events with specific temporal characteristics successfully keeping track of their values while other events may also occur. In our implementation, the use of a dedicated Event-id module (see Figure 4) enables the correct association between events and times, filtering out irrelevant external stimuli.

Overall, the following points summarize the differences between the *how-long* and *when* modes of operation in the model:

- *How-long* and *when* assume respectively a linear and a logarithmic representation of time, therefore accomplishing to measure durations of different scales (up to 50 simulation steps for *how-long* vs. up to 1000 simulation steps for *when*).
- The *how-long* mechanism aims at counting the time filled with the occurrence of an event, while the *when* mechanism counts time that is not any more in direct link to the given event.
- *How-long* results into the final encoding of a static duration value in memory, while *when* assumes an ever-changing, dynamic representation of past times relative to the moving present.

Currently, the model exhibits two limitations which, at the same time, offer two important strands for future work. The first regards the representation of far distant times which ordinary models address by assuming processes that can increase without limit (Miall, 1989; Matell and Meck, 2004; Large, 2008; Simen et al., 2011). Despite the fact that such unbounded processes can hardly provide a realistic explanation of time perception (Staddon and Higa, 1999), they do not address multiscale time perception that is innate for humans. Interestingly, the newly introduced DDM (Simen et al., 2013) model which uses adapting pulse rates to measure time intervals could provide a means for implementing multi-scale time perception, assuming the future implementation of a time abstraction mechanism (i.e., I am only aware that I moved to a new city 6 months ago, but I do not know how many seconds or minutes have passed since then). In the present work, the use of sigmoid activation functions in the output neurons of the model does not fully comply with the representation of far distant times. Sigmoid functions produce outputs in the range [0, 1], therefore they are not appropriate for approximating logarithmic times greater than one. To compensate this limitation we plan to implement multi-scale time perception in the cognitive system, similar to (Staddon and Higa, 1999). Each time scale will be implemented as a logarithmic function with a basis of a second, a minute, an hour and so on (i.e., log_sec_, log_min_, log_hour_, etc.). An event that approximates the maximum sigmoid value of one in a given scale will “jump” to the next scale, starting from a relatively low value which will gradually increase to one being ready for a new “jump” and so on.

The second direction for advancing the model regards the perception of not only past, but also future times. This important addition will pave the way for investigating long-term planning, self-projection to the future, imagination and other high level cognitive skills which are currently unattainable in artificial systems. Similar to past perception, we plan to implement future time perception following the assumption of logarithmic multi-scale times. Future perception will look like past perception, horizontally flipped with respect to zero-time that represents “now”. The composite model will be able to perceive future (expected) events approaching the present, be part of reality (occur) and then moved to the past (memorized).

The embodiment of the model into a robotic system and its practical application in real life has revealed some particularly challenging issues for artificial temporal cognition. So far we assume that experienced events are assigned ids in a periodic manner, i.e., in the form 1,2,3,4,5,6,1,2,3,4…and so on, and thus their temporal characteristics are circularly encoded in the relevant output modules in short term memory. As new events are experienced by the agent, previous events should be either deleted or transferred to long-term memory. The details of this mechanism remains an open research issue, however by mixing elapsed time and the attention devoted to the event we have been able to implement rough criteria that facilitate decision making with respect to the handling of past events. Currently we use a simple Data Base system to encode past events in LTM, but we are also investigating neural representations that will enable abstracting and encoding events in the form of episodes.

## Conclusions

Our perception and consideration of time, is key in determining how we behave and in the decisions we make. Besides the increasing research interest that is recently devoted on temporal cognition there not much studies linking the *how-long* and *when* aspects of perceived events. Both of these aspects are fundamental for the rich and meaningful perception of the environment. The present work considers a memory representation perspective to link short- and long-term time perception, accomplished by using a single timing source to perceive both event-specific and event-irrelevant times.

The broader vision of our research aims at time-aware artificial autonomous systems. The particularly promising results of the current work suggest that the proposed timing model can be the basis for implementing artificial systems that successfully interact with humans for the collaborative accomplishment of short- and mid-term goals.

### Conflict of interest statement

The authors declare that the research was conducted in the absence of any commercial or financial relationships that could be construed as a potential conflict of interest.

## Footnotes

^1^We have followed both modeling assumptions in our work and we have successfully implemented distant time models assuming either a linear or a logarithmic representation of time. The current paper demonstrates only the logarithmic approach but it is straightforward to adapt the evolutionary procedure with the assumption of a linear time.
